# Supplementary material for: The Gut Bacteria Dysbiosis Contributes to Chronic Graft-Versus-Host Disease Associated With a Treg/Th1 Ratio Imbalance
Source: Front Microbiol. 2022 Sep 8;13:813576. doi: 10.3389/fmicb.2022.813576 (PMC9493085; doi:10.3389/fmicb.2022.813576)
Supplement: Supplementary file 1 [file Data_Sheet_1.zip › P101SC18090073-01-B1-3-4_result/05.WebShow/WebShow.pdf]

WebShow 交互式网页版展示说明

北京诺禾致源生物信息科技有限公司

科技服务部微生物事业部

目录

一、网页版 **OTU** 热图（**OTU Heatmap**）结果查看和解读 2
1. 文件结构 2
2. 结果查看步骤 2
3. 结果解读 3
二、网页版物种注释堆积图（**Taxa summary**）结果查看和解读 3
1. 文件结构 3
2. 结果查看步骤 3
3. 结果解读 5
三、网页版物种注释可视化圈图（**Krona**）结果查看和解读 6
1. 文件结构 6
2. 结果查看步骤 6
3. 结果解读 6
四、网页版 **Alpha** 多样性（**Alpha Rarefaction**）结果查看和解读 7
1. 文件结构 7
2. 结果查看步骤 7
3. 结果解读 8

一、网页版 OTU 热图（OTU Heatmap）结果查看和解读

1. 文件结构

网页版展示结果，见：result/Webshow/OTU_heatmap/sorted_otu_table.html；

网页配置文件，见：result/Webshow/OTU_heatmap/js/*。

2. 结果查看步骤

2.1 用浏览器打开 sorted_otu_table.html 文件，得到如下显示：

Filter by Counts per OTU: [ ] [Sample ID] [Taxonomy]

2.2 在输入框中输入筛选数字，点击 Sample ID 或 Taxonomy 按钮选取不同方式对筛选方式进行展示

a. 筛选数字的选择（Filter by Counts per OTU）：此处的筛选条件是按照每个 OTU 在各个样品中的已注释 Tags 数目的总数进行筛选，默认的筛选值是 5，但其实这个阈值并没有很推荐的经验值，选取的条件建议依据老师的研究环境和已有经验，以及希望解释的生物学问题筛选即可。如果老师重点关注感兴趣的物种，可将阈值尽量设小以展示更多的结果；而如果老师比较关注高丰度的物种，可适当将阈值调高，在展示时将低丰度物种过滤掉。

b. 点击“Sample ID”时，呈现的结果是每个样本在每个 OTU 上的 Tags 数目，此时鼠标每指到一个 OTU 时，就会显示该样本在该 OTU 中注释到的物种信息。

#OTU ID	OTU_1	OTU_2	OTU_3	OTU_4	OTU_5	OTU_6	OTU_7	OTU_8	OTU_9	OTU_10
test1	399	487	655	394	479	140	364	204	15	333
test2	360	311	467	134	307	171	98	40	16	354
test3	396	329	503	338	261	229	114	194	22	292
test4	300	496	521	454	308	36	154	175	30	207
test5	391	296	533	92	177	90	66	67	6	240
test6	384	317	521	117	194	124	56	157	9	261
test7	380	389	627	61	206	127	50	103	12	300
test8	318	410	591	184	232	103	78	140	11	254
test9	357	369	580	145	440	262	480	105	10	299
test10	331	508	682	289	274	126	125	203	8	230
test11	388	365	575	186	505	169	187	146	19	274
test12	294	441	664	227	331	218	228	138	29	224

c. 点击“Taxonomy”时，则会将所有样本的所有 OTU 注释物种信息显示出来，您可以同时按住 ctrl+F，在左下角会显示出搜索框，输入您所关心的菌种名或属名等等信息，可以看到该菌在样本中被注释到的情况。

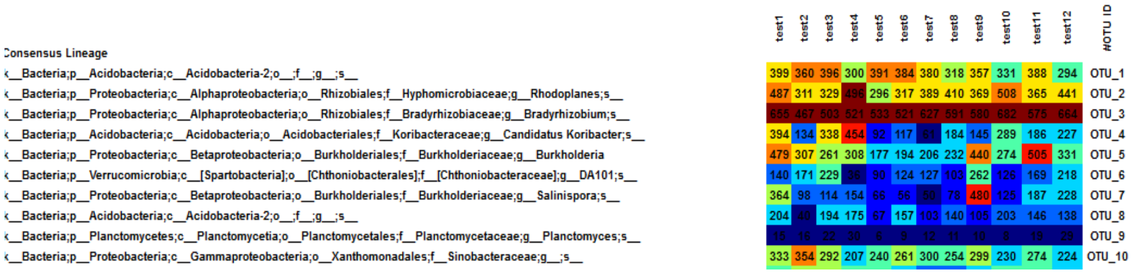

3. 结果解读

Heatmap 数字表示某个样品中某个 OTU 或分类所包括的 Tags 数目，每一对样品名和 OTU 对应的网格中的颜色表示该 OTU 或分类所包括的序列（Tags）数目水平，蓝色表示低，红色表示高。 OTU 的编号与结果文件中的 OTU_table 中编号一致，按照 reads 数目的由多到少排序。详细信息可以参考 http://qiime.org/1.3.0/tutorials/tutorial.html#make-otu-heatmap，简要可以参考下图。

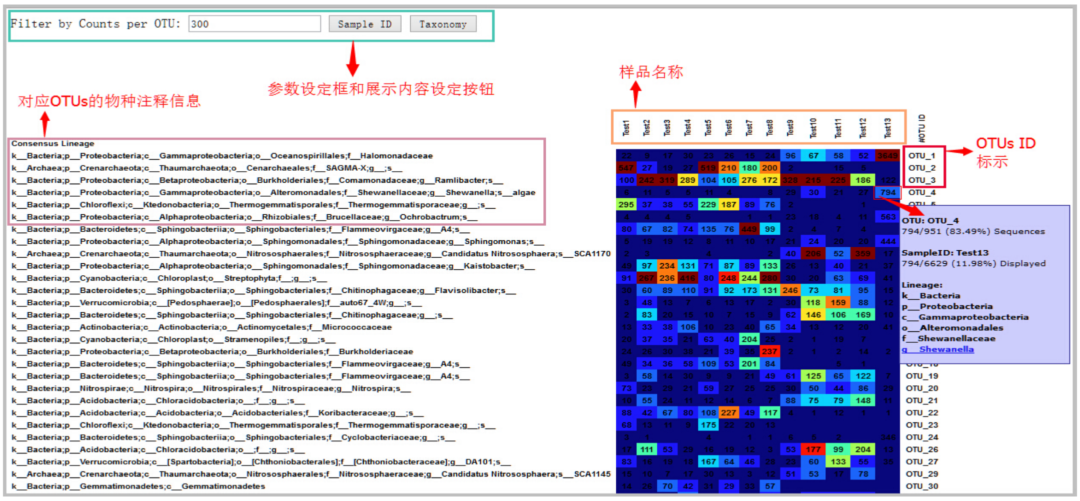

二、网页版物种注释堆积图（Taxa summary）结果查看和解读

1. 文件结构

网页版展示结果，见：result/Webshow/taxa_charts_html/{area/bar/pie}_charts.html；

注释结果的堆积图，见：result/Webshow/ taxa_charts_html /charts/*.{pdf /png}；

网页配置文件，见：result/Webshow/ taxa_charts_html /js/*和 result/Webshow/ taxa_charts_html /css/*。

2. 结果查看步骤

2.1 用浏览器打开{area/bar/pie}_charts.html 文件，得到不同分类层级的柱状/面积/饼图。

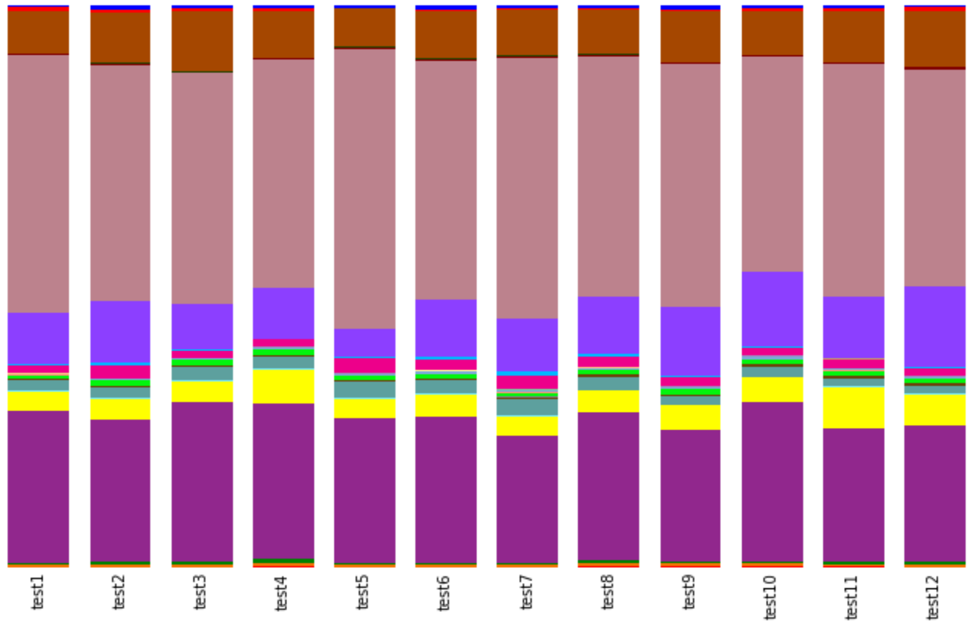
test1
test2
test3
test4
test5
test6
test7
test8
test9
test10
test11
test12

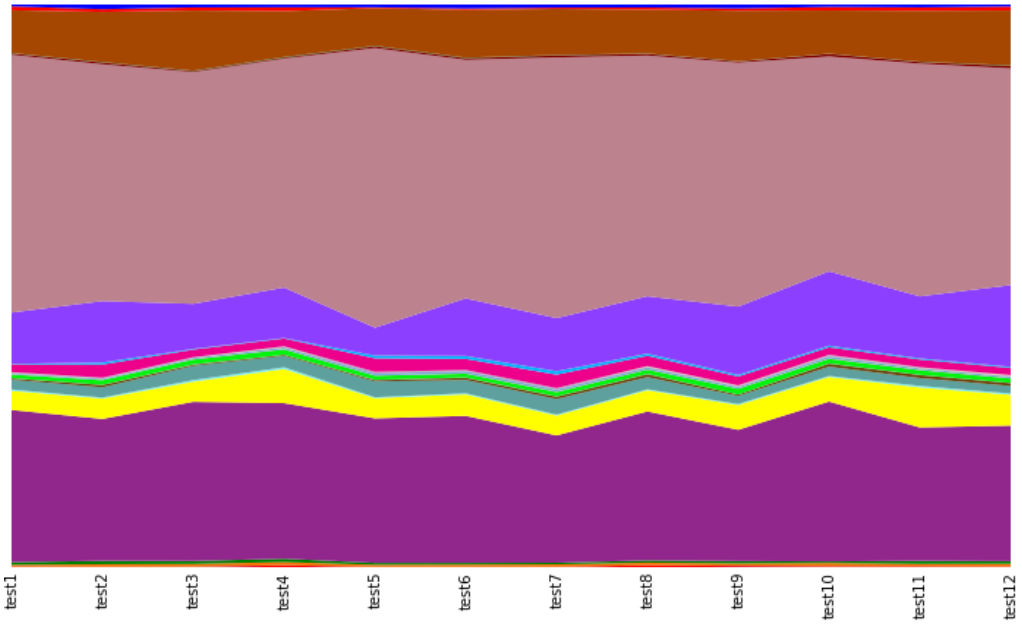
test1
test2
test3
test4
test5
test6
test7
test8
test9
test10
test11
test12

: 12.0 (of 12.0) from 20 categories displayed, including 0.0409581489246 from 13 categories ('All Other Categories')

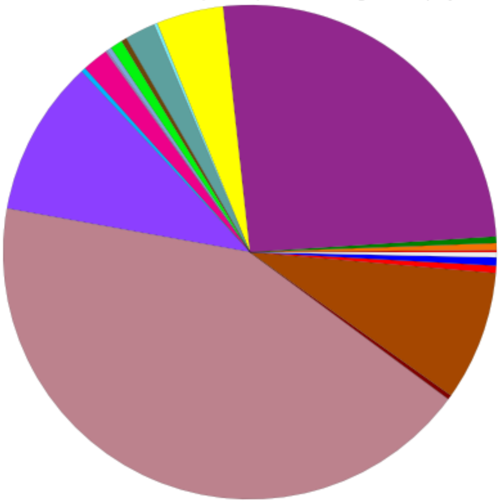

2.2 每个图下配有统计信息，展示该分类层级上的具体注释结果和在样品中的百分含量信息。

View Table (.txt)

		Total		test1	test2	test3	test4	test5	test6	test7	test8	test9	test10	test11	test12
Legend	Taxonomy	count	%	%	%	%	%	%	%	%	%	%	%	%	%
	k__Archaea;p__Crenarchaeota	0	0.1%	0.1%	0.1%	0.1%	0.3%	0.1%	0.1%	0.1%	0.3%	0.2%	0.1%	0.2%	0.1%
	k__Archaea;p__Euryarchaeota	0	0.0%	0.0%	0.0%	0.0%	0.0%	0.0%	0.0%	0.0%	0.0%	0.0%	0.0%	0.0%	0.0%
	k__Bacteria;Other	0	0.5%	0.3%	0.5%	0.5%	0.6%	0.4%	0.4%	0.5%	0.4%	0.4%	0.6%	0.4%	0.5%
	k__Bacteria;p__AD3	0	0.4%	0.5%	0.6%	0.5%	0.6%	0.3%	0.3%	0.3%	0.4%	0.4%	0.3%	0.5%	0.5%
	k__Bacteria;p__Acidobacteria	3	25.7%	27.1%	25.2%	28.3%	27.7%	25.7%	26.1%	22.6%	26.5%	23.3%	28.4%	23.7%	24.1%
	k__Bacteria;p__Actinobacteria	1	4.4%	3.4%	3.6%	3.6%	6.1%	3.6%	3.8%	3.5%	3.8%	4.4%	4.4%	7.1%	5.5%
	k__Bacteria;p__Armatimonadetes	0	0.2%	0.2%	0.2%	0.3%	0.3%	0.2%	0.2%	0.2%	0.2%	0.2%	0.2%	0.3%	0.2%
	k__Bacteria;p__BRC1	0	0.0%	0.0%	0.0%	0.0%	0.0%	0.0%	0.0%	0.0%	0.0%	0.0%	0.0%	0.0%	0.0%
	k__Bacteria;p__Bacteroidetes	0	2.0%	1.8%	1.8%	2.5%	2.0%	2.8%	2.4%	2.8%	2.2%	1.4%	1.7%	1.4%	1.3%
	k__Bacteria;p__Chlamydiae	0	0.4%	0.2%	0.4%	0.2%	0.2%	0.2%	0.4%	0.4%	0.5%	0.2%	0.5%	0.5%	0.5%
	k__Bacteria;p__Chlorobi	0	0.1%	0.1%	0.0%	0.1%	0.0%	0.1%	0.1%	0.1%	0.1%	0.0%	0.1%	0.0%	0.0%
	k__Bacteria;p__Chloroflexi	0	0.8%	0.6%	0.8%	0.9%	0.9%	0.7%	0.7%	0.8%	0.8%	1.0%	0.8%	0.8%	0.8%
	k__Bacteria;p__Cyanobacteria	0	0.0%	0.0%	0.0%	0.0%	0.0%	0.0%	0.0%	0.1%	0.0%	0.0%	0.1%	0.0%	0.0%
	k__Bacteria;p__Elusimicrobia	0	0.3%	0.2%	0.2%	0.2%	0.3%	0.4%	0.4%	0.4%	0.4%	0.3%	0.4%	0.3%	0.3%
	k__Bacteria;p__FCPU426	0	0.1%	0.0%	0.1%	0.0%	0.1%	0.0%	0.0%	0.1%	0.1%	0.1%	0.1%	0.1%	0.1%
	k__Bacteria;p__Fibrobacteres	0	0.0%	0.0%	0.0%	0.0%	0.0%	0.0%	0.0%	0.0%	0.0%	0.0%	0.0%	0.0%	0.0%
	k__Bacteria;p__Firmicutes	0	0.1%	0.1%	0.1%	0.1%	0.1%	0.2%	0.1%	0.2%	0.1%	0.1%	0.1%	0.1%	0.1%
	k__Bacteria;p__Fusobacteria	0	0.0%	0.0%	0.0%	0.0%	0.0%	0.0%	0.0%	0.0%	0.0%	0.1%	0.0%	0.0%	0.1%
	[illegible]	[illegible]	[illegible]	[illegible]	[illegible]	[illegible]	[illegible]	[illegible]	[illegible]	[illegible]	[illegible]	[illegible]	[illegible]	[illegible]	[illegible]

3. 结果解读

3.1 图形解读

图中横坐标是样品名，纵坐标表示各物种在样品中的相对丰度，将鼠标移动至图中任一颜色填充区域即可得到对应的物种注释结果和丰度信息。

3.2 图下统计解读

图下统计结果第一列给出了图例颜色，第二列给出该分类的注释结果，第三大列的 total 方格内给出了该分类在所有样品中的相对丰度统计情况，后面依次给出了该分类在不同样品中的存在情况和丰度分布，如果该分类存在于样品中，则用与图例同色的方框将对应丰度信息框起来，如果该分类未出现在这个样品中，则方框不着色。

对于具体的统计信息，%对应的方格内的数字反映了该分类在各个样品中的相对丰度情况，代表在所有样本中某一物种所占的比例；count 是对相对丰度取整的结果，对单个样品而言，相对丰度值大于 0.5 取值 1，小于 0.5 取值 0，相应地，Total 栏下的 count 值，是所有样品相对丰度之和四舍五入的值。

英文原文如下：The counts displayed pertain to either relative or absolute values depending on your selection from summarize_taxa.py. For relative values, the numbers are converted to interger, so counts below 0.5 appear as 0.

3.3 详细信息可参考：http://qiime.org/1.2.1/tutorials/taxonomy_summaries.html。

三、网页版物种注释可视化圈图（Krona）结果查看和解读

1. 文件结构

网页版 Krona 展示结果，见：result/Webshow/all_rep_set_tax_assignments.krona.html。

2. 结果查看步骤

用浏览器打开 all_rep_set_tax_assignments.krona.html 文件，得到每个样品的注释结果圈图展示如下：

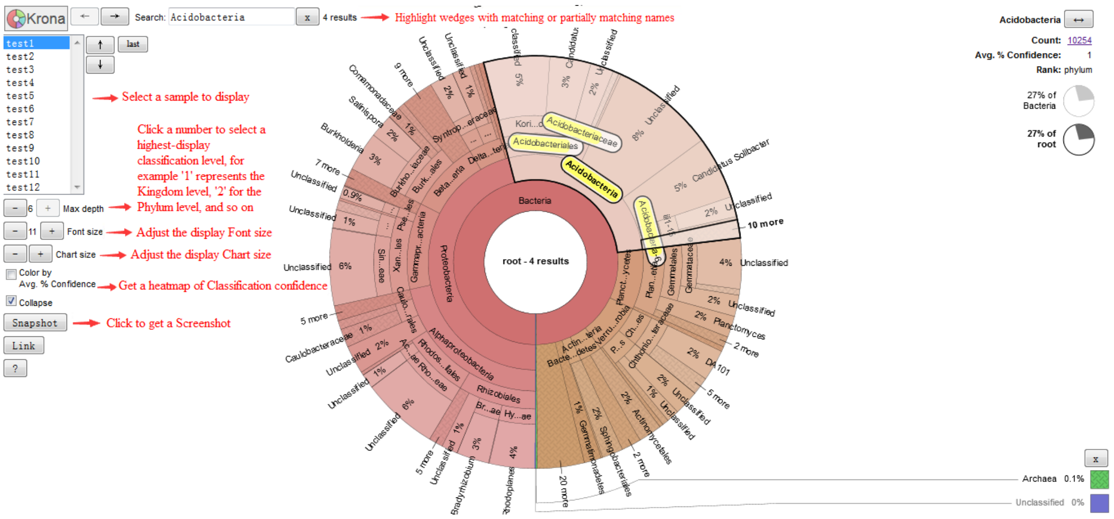

3. 结果解读

3.1 图形解读

在 Krona 展示图中，由内至外的圆圈依次代表由界至种的分类层级，通过调整 Max depth 对应的数字（1 代表界水平，2 代表门水平，等）可调节展示物种的最高分类级别。如果想具体看图中某一特定物种的丰度信息，可用鼠标选中某一物种，此时在右上角会对应出现该物种的分类级别、物种在上一分类级别中所占比例和在全部注释结果中所占比例情况。此外，对于一些痕量物种，由于其丰度过低，物种展示的扇形过小，将用箭头指向右侧空白处并图例标注对应颜色。

对图的展示有初步的了解后，可点选左侧的下拉菜单选择要展示的样品，得到选中样品的注释信息。选好要展示的样品后，在 Search 框中填入部分或全部物种名，可按输入文字进行对应检索，检索到的文字标黄显示。

图形的输出，可用 Font size 调整字体大小，Chart size 调整图片大小，Color byAvg. % Confidence 可对注释结果按置信度进行着色等，调整图片合适后可直接右键对图片另存，也可用 Snapshot 导出后另存为 SVG 矢量图。

3.2 详细信息可参考 Krona 网站上的介绍：
http://sourceforge.net/p/krona/wiki/Browsing%20Krona%20charts/。

四、网页版 Alpha 多样性（Alpha Rarefaction）结果查看和解读

1. 文件结构

网页版 Alpha 多样性稀释曲线展示结果，见：
result/Webshow/alpha_rarefaction_plots/rarefaction_plots/rarefaction_plots.html；

不同 Alpha 多样性指数的稀释曲线图，见：
result/Webshow/alpha_rarefaction_plots/average_plots，其中*SampleID.png 是全样品稀释曲线图，*Description.png 是分组稀释曲线图；

全部作图数据，见：
result/Webshow/alpha_rarefaction_plots/average_plots/average_tables/*；

网页配置的图形文件，见：result/Webshow/alpha_rarefaction_plots/html_plots/*。

2. 结果查看步骤

2.1 用浏览器打开 rarefaction_plots.html 文件，得到如下显示：

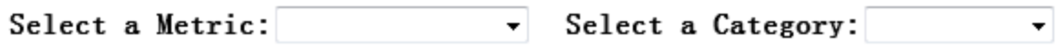

2.2 在输入框的下拉菜单中选择一种多样性指数（chao1, observed_species, shannon, simpson, goods_coverage)和一种样品展示方式(SampleID 表示按样品名展示，Description 表示按分组展示）显示图片。

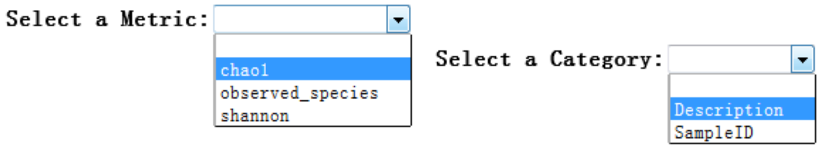

2.2 通过勾选相图例（Legend）中对应的样品名调整该样品是否显示在图中。

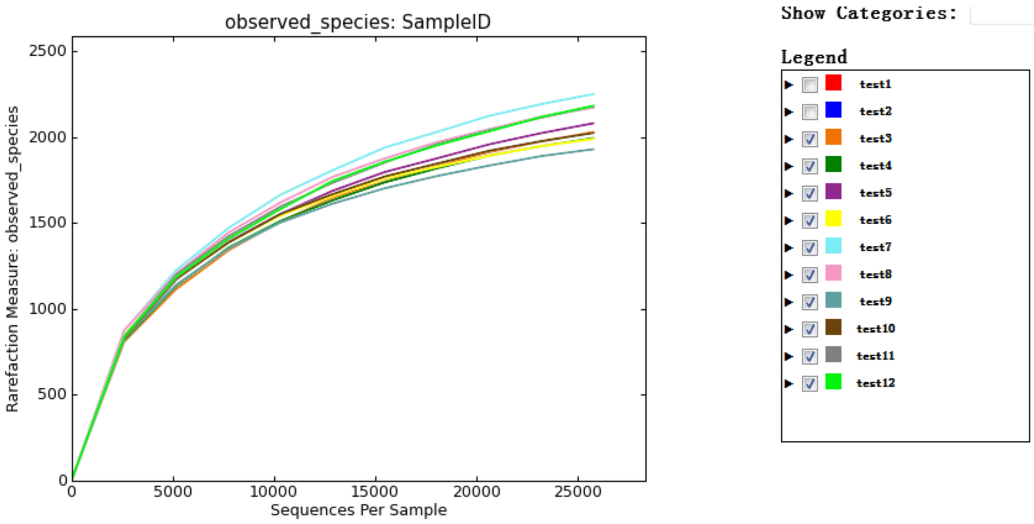

2.2 每个图下配有统计信息，展示在不同抽取深度对应的指数值的计算结果，其中分组数据提供组内计算均值（Ave.）和误差（Err. ）。

a. 单样品稀释曲线数据

SampleID	Seqs/Sample	chao1 Ave.	chao1 Err.	observed_species Ave.	observed_species Err.	shannon Ave.	shannon Err.
test1	10.0	51.800	nan	9.900	nan	3.302	nan
test1	2584.0	1402.305	nan	779.300	nan	8.606	nan
test1	5158.0	1796.039	nan	1100.200	nan	8.797	nan
test1	7732.0	1972.939	nan	1318.200	nan	8.895	nan
test1	10306.0	2113.318	nan	1491.000	nan	8.945	nan
test1	12880.0	2197.266	nan	1626.800	nan	8.979	nan
test1	15454.0	2240.920	nan	1733.400	nan	8.999	nan
test1	18028.0	2242.481	nan	1810.000	nan	9.013	nan
test1	20602.0	2243.999	nan	1887.300	nan	9.032	nan
test1	23176.0	2253.805	nan	1951.900	nan	9.038	nan
test1	25750.0	2231.452	nan	2000.900	nan	9.047	nan
test2	10.0	51.800	nan	9.900	nan	3.302	nan
test2	2584.0	1556.726	nan	866.300	nan	8.907	nan
[illegible]	[illegible]	[illegible]	[illegible]	[illegible]	[illegible]	[illegible]	[illegible]

b.分组样品稀释曲线数据

Description	Seqs/Sample	chao1 Ave.	chao1 Err.	observed_species Ave.	observed_species Err.	shannon Ave.	shannon Err.
group1	10.0	50.750	1.819	9.850	0.087	3.292	0.017
group1	2584.0	1462.041	57.801	817.850	31.248	8.747	0.107
group1	5158.0	1834.405	65.777	1142.300	45.338	8.950	0.118
group1	7732.0	2028.397	78.970	1370.350	56.108	9.050	0.119
group1	10306.0	2171.090	84.037	1537.425	57.087	9.099	0.121
group1	12880.0	2241.073	81.936	1669.825	59.037	9.130	0.120
group1	15454.0	2303.579	92.852	1775.900	65.333	9.152	0.123
group1	18028.0	2306.178	91.131	1859.275	66.515	9.167	0.121
group1	20602.0	2316.805	83.761	1935.425	65.232	9.182	0.119
group1	23176.0	2306.365	84.369	1998.575	69.460	9.191	0.121
group1	25750.0	2289.626	80.790	2049.750	71.222	9.200	0.122
group2	10.0	49.400	3.487	9.825	0.109	3.287	0.022
group2	2584.0	1534.135	86.886	856.250	21.066	8.916	0.031
group2	5158.0	1907.963	50.526	1195.975	22.422	9.119	0.026

3. 结果解读

3.1 图形解读

图中横坐标为从某个样品中随机抽取的序列条数，纵坐标为基于该测序条数计算所得的某一多样性指数值，不同的样品使用不同颜色的曲线表示。

3.2 图下统计解读

图下注释部分给出了具体的作图数据，选择单样品展示模式（选择 SampleID）时，图下展示的统计结果的每一行中包含以下信息：第一列是样品名；第二列是从某个样品中随机抽取的序列条数；第三列是该深度下计算所得的某一个指数的值；第四列是计算误差，由于是单样品不存在误差的说法，因此这一列全部为 nan；第五列是第二个多样性指数的计算值，等等，以此类推。

选择分组展示模式（选择 Discription）时，图下统计结果与前述单样品格式相同，区别的是第三列（和第五列、第七列等）处的数字为该组所有样品的该指数值的均值，相应的下一列为该组所有样品的指数的标准差。

详细信息可参考：http://qiime.org/1.3.0/tutorials/tutorial.html。

3.3 序列抽取原则

采用 Qiime 软件（Version 1.7.0）默认参数，设定最小抽取序列数为 10，最大抽取数为所有样品中最少的对应序列条数，步长为（最大抽取数-最小抽取数）/10，每一步重复取样 10 次。

详细信息可参考：http://qiime.org/scripts/alpha_diversity.html。
